# Down-Regulation of Cysteine-Glutamate Antiporter in ALDH1A1 Expressing Oral and Breast Cancer Stem Cells Induced Oxidative Stress-Triggered Ferroptosis

**DOI:** 10.7150/jca.89429

**Published:** 2024-10-07

**Authors:** Ravi Shankar Bellala, Prasanthi Chittineedi, Sungey Naynee Sánchez Llaguno, Juan Alejandro Neira Mosquera, Gooty Jaffer Mohiddin, Santhi Latha Pandrangi

**Affiliations:** 1 Onco-Stem Cell Research Laboratory, Dept of Life Sciences, School of Science, GITAM (Deemed to Be) University, Visakhapatnam-530045, India.; 2 Department Of Life Sciences and Agriculture, Armed Forces University-Espe, Santo Domingo 230101, Ecuador, South America.

**Keywords:** Cancer Stem Cells, Ferroptosis, Ferritin, ALDH1A1, Cysteine-Glutamate Antiporter

## Abstract

**Background:** Sulfasalazine, an xCT inhibitor, is being used as a repurposed antineoplastic drug to induce ferroptosis. Ferroptosis is a regulated necrotic cell death pathway that is dependent on iron reserves. Interestingly, cancer stem cells (CSCs) that are regarded as major drivers of resistance to conventional therapies accompanied with tumor relapse and recurrence have bulk amount of iron reserves in the form of ferritin. This suggests that inducing ferroptosis might disrupt stemness and drug-resistant mechanisms in cancer stem cells, thereby reducing the risk of drug-resistance, cancer recurrence, and relapse.

**Materials & Methods:** In the present study, ALDH1A1 expressing oral (OCSCs) and breast (BCSCs) cancer stem cells were sorted and used to investigate the role of sulfasalazine to induce ferroptosis. To check the self-renewability of CSCs spheroid formation, assay was performed and the resultant CSCs were treated with sulfasalazine (SAS) and subjected to gene expression analysis RT-PCR and flow cytometry. FACS was performed to check stem cell marker expression, cell cycle arrest, and apoptosis.

**Results:** Our results suggest that the cells showed a gradual increase in sphere formation till S3 in the case of OCSCs and S2 in the case of BCSCs, with a gradual decrease in sphere-forming efficiency from the respective generations. When treated with 0.6mM SAS, these cells induced ferroptosis by downregulating stem cell markers like ALDH1A1, SLC7A11, ferritin, and GPx-4 with a concomitant increase in transferrin and STEAP-3. Flow cytometry studies revealed that the cells have undergone mitochondrial dysfunction characterized by loss of membrane potential and the cell cycle progression was halted in the G2/M phase.

**Conclusion:** In the present study, we demonstrate that SAS potentially induced ferroptosis accompanied with oxidative stress in both OCSCs as well as BCSCs by lowering GPx-4 activity, a key enzyme that scavenges the products produced as a result of oxidative stress.

## 1. Introduction

Cancer is a disorder that plays a major role in death statistics worldwide. This ailment could be regarded as cellular impairment caused due to mutations in certain genes involved in the cell cycle and cell signaling that regulates cell growth, proliferation, and death 1. It is a condition where the cells grow and multiply abruptly. Usually, in a healthy person, damaged cells would be arrested by specific cell cycle checkpoints, thereby triggering apoptosis 2. For instance, p53 is the major cell cycle checkpoint that checks for genome stability and triggers cell cycle arrest, cellular senescence, and/ or cell death, usually apoptosis in response to DNA damage 3, in malignant cells, p53 carries loss-of-function mutation and the mutant p53 serves as a dominant-negative inhibitor against wild-type p53 leading to dysregulation in cell proliferation and cell death processes Although chemotherapy, radiotherapy, and surgery have been a part of cancer treatment, resistance to therapy and cancer recurrence and relapse is showing a major impact on cancer incidence and mortality rate. The possible reason for therapy resistance and recurrence might be due to the presence of CSCs 4. CSCs are regarded as tumor-initiating cells which does not respond to drugs and radiation, as they stay in the latent phase of the cell cycle and grow very slowly, constituting the leading cause of tumor relapse/recurrence 5, 6.

Breast cancer occupies first place in terms of cancer incidence in females, while oral cancer stands sixth in terms of the rate of cancer incidence, particularly in men, with a high relapse ratio 7, 8 Literature suggests that the presence of a CSC niche in the tumor microenvironment is the sole reason for tumor relapse/recurrence 5. Aldehyde Dehydrogenase 1 family member A 1 abbreviated as ALDH1A1 which is a stemness marker is NADP+ dependent cytosolic homotetrameric enzyme, which is ubiquitously circulated in adult organs such as the kidney, brain, testis, lens, eye, lungs, liver, and retina 9, 10. There are three major isoforms for ALDH1A1 viz., ALDH1A1, ALDH1A2, and ALDH1A3 11. ALDH1A1 is intricated in the biology of both normal stem cells (NSCs) as well as in CSCs by mediating cell differentiation and drug resistance 12. Additionally, this enzyme is involved in the major pathway of alcohol metabolism, thereby detoxifying wide varieties of endogenous and exogenous aldehydes into weak carboxylic acids 10, 13. ALDH1A1, which is localized in the nucleus, plays a crucial role in inhibiting ROS, which is essential for inducing ferroptosis. Ferroptosis is a newly-emerged cell death pathway that is dependent on iron reserves 14. Surprisingly, CSCs have bulk iron reserves, and hence they are more sensitive to ferroptosis 15, 16. Studies have demonstrated that elevated expression of ALDH1A1 is associated with poor prognosis 17. Numerous treatment regimens are employed to treat cancer patients, though these regimens are ineffective to target CSCs. This is because of their ability of self-renewal, development of therapy resistance, and apoptosis resistance. However, recent evidences suggest that both cancer and CSCs tend to accumulate more iron and store it in the form of ferritin. In fact, CSCs, when compared with cancer cells, have more ferritin levels. Hence targeting iron metabolism resulting in elevation of oxidative stress leads to a novel death mechanism termed as ferroptosis. Ferroptosis, as the name suggests, is an iron-dependent cell death mechanism that is induced in the absence of caspase and one of the few pathways to which CSCs might be sensitive. The present study focused on inducing oxidative stress, which triggers ferroptosis via ferritin degradation in ALDH1A1 oral and breast cancer stem cells.

## 2. Materials and Methods

### 2.1. Culture and maintenance of oral cancer and breast cancer cells

To perform the present study, the oral cancer cell line (UCSF-OT-1109) and breast cancer cell line (MCF-7), MCF-10A which is regarded as non-tumorigenic control were procured from ATCC and NCCS, respectively. The cell lines were maintained in DMEM media (Invitrogen, Carlsbad, CA, USA), supplemented with heat inactivated fetal bovine serum (Gibco) (10%), Pen Strep (10%) (Invitrogen, Carlsbad, CA, USA), and insulin (5µg/mL) (Sigma-Aldrich, St. Louis, MO, USA).

### 2.2. Quantification of CSCs using ALDEFLOUR assay and flow cytometry

ALDEFLOUR assay was used to quantify and sort CSCs present in the cultured cell lines based on the presence of the aldehyde dehydrogenase (ALDH1A1) marker. ALDEFLOUR assay was performed as done in the previous studies 13. Briefly, single cells generated from the cell culture were incubated in Aldeflour assay buffer for 45 mins at 37^0^ C. As a negative control, a fraction of cells was incubated under identical conditions in the presence of an ALDH inhibitor, diethylamino benzaldehyde (DEAB). The sorting gates were established using the negative control. Flow cytometry was conducted using FACS Aria II (Becton Dickinson, Franklin Lakes, NJ, USA) at NIAB (National Institute of Animal Biotechnology), Hyderabad.

### 2.3. Spheroid formation assay

Tumor spheroids were grown in serum-free media supplemented with bFGF (Gibco), B27 (Gibco), and human recombinant insulin (Sigma) under low adherence conditions, as described by Dontu *et al.* (2003). The sorted out ALDH1A1/bright cells (termed as Spheroids Forming Units-SFUs) and the ALDH1A1 cells were plated in 6-well ultra-low attachment plates (Corning, MA) in triplicates for the formation of primary spheroids at the density of 2*10^3^ cells/well. The resulting primary spheroids after 10 days (termed as S1 spheroids) were collected by centrifugation at 500 rpm for 3 mins. Both oro- and mammo-spheroids were counted microscopically under a manually-prepared quadrant grid. Briefly, the spheroids were mechanically dissociated using a Pasteur pipette, passed through a 45nm sieve (Beckton Dickinson, Franklin Lakes, NJ, USA) followed by enzymatic dissociation using accutase. The spheroids were washed twice with 1X PBS and treated with 1mL of 1X accutase for 2mins while swirling gently. The enzyme was then discarded, 2mL of fresh media was added and the treated spheroids were passed through the Pasteur pipette gently to obtain single cell suspension. The dissociated cells were sorted again using FACS. Later, oral and breast CSCs' efficiency in generating spheres was measured. The total number of spheroids obtained were counted, and SPE was calculated using the formula;


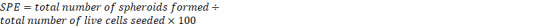
.

### 2.4. Enrichment of ALDH1A1/bright CSCs

To test the functional definition of stem cells to self-renew, these SFUs enriched for oral and breast CSCs were again plated after live cell count for the spheroid formation and were subjected to serial passaging every 10th day, leading to the generation of S2, S3, S4 spheroids, and so on. The spheroids were counted after every passage, and the images were recorded using the EVOS FL Imaging system (Thermo Fischer Scientific).

### 2.5. MTT cell proliferation assay

To study the cell death kinetics, OCSCs and BCSCs were treated with increasing concentrations of sulfasalazine for 24 h, and cell survival is evaluated by MTT assay. Wells containing only the cells and medium with no drug added were taken as controls. After incubation, the cells were fed with 200 μl of fresh medium, 50μl of MTT (Sigma-Aldrich, St. Louis, MO, USA) solution (2 mg/ml phosphate-buffered saline) was added, and the plates were incubated for 3 h. After incubation, the media containing MTT solution was removed, and the formazan crystals were dissolved by adding 200 μl of DMSO to each well. The absorbance was read at 570 nm for each well immediately using a multi-well spectrophotometer. The absorbance of control cells was taken as 100% viability and the values of treated cells were calculated as a percentage of the controls. Each value is the mean of eight wells with a standard deviation (SD).

### 2.6. Pharmacological inhibition of SLC7A11

To study the effect of self-renewal ability and the ability of cells to induce ferroptosis, drug treatment with the obtained IC50 value was performed on 80% confluent cells. Briefly, cells were grown in 35mm Petri-plates and incubated in 37^0^C. Once the cells were 80% confluent, OCSCs were treated with 0.6mM sulfasalazine while BCSCs cells were treated with 0.3mM sulfasalazine for 24 hours. The drug-treated cells were used for further analysis.

### 2.7. Expression analysis

#### 2.7.1. RT PCR analysis of pharmacologically-inhibited ALDH1A1/bright OCSCs

To analyze the gene expression of various ferroptosis markers in ALDH +ve CSCs treated with SAS (0.6mM for OCSCs and 0.3mM for BCSCs) RT-PCR analysis was performed. Initially, cells were seeded in 25-cm^2^ culture flasks (3.5 × 10^5^ cells/4 ml medium). Following treatments, the cells were collected, washed twice in ice-cold phosphate-buffered saline (PBS), and total RNA was extracted using TriZol (Invitrogen, Carlsbad, CA, USA). The isolated RNA was immediately converted to cDNA with the help of a cDNA conversion kit (Applied Biosystems) according to the manufacturer's instructions. The cDNA was used to analyze the expression of ferroptosis makers in drug-treated cells using the SYBR green master RT-PCR mix (Eurogentec UF RSMT B0101). The reaction mixture was prepared according to the manufacturer's instructions and 10uL of cDNA sample was added. The reaction mixture containing cDNA was then placed in a thermal cycler and the expression was analyzed. GAPDH was used as an internal control. Table 1 represents various sets of primers used in the present study.

#### 2.7.2. Immunofluorescence

To analyze the cellular expression of SLC7A11 and OCT-4, in SAS-treated and untreated OCSCs and BCSCs immunofluorescence (IF) was performed. Briefly, the cells were seeded on the coverslips. 70% confluent cells were treated for 24 hours with 0.6mM (OCSCs) and 0.3mM (BCSCs) sulfasalazine. Treated cells were washed twice with PBS, fixed with 4% paraformaldehyde for 30 mins, and permeabilized with 0.2% triton-X for 10 mins. The cells were blocked with 10% BSA for 30 mins, incubated with primary antibody against SLC7A11 and OCT4 overnight at 4^0^C, and incubated with secondary antibody (Catalog # 32460) for 1 hr. The cells were washed twice with PBS after each step. Finally, nuclear staining using DAPI was done and the fluorescent images were captured using EVOS FLc fluorescent microscope.

### 2.7.3 Western blot analysis

To analyze the protein expression of NANOG western blot was performed. SAS treated and untreated cells of both BCSCs and OCSCs were scrapped from the culture plate, lysed using HEPES lysis buffer to extract the cytoplasmic proteins. 60ug of the extract protein was mixed with equal volume of lamaelli buffer and heated for 5 mins at 95^0^C.The proteins were separated using 8% polyacrylamide gel and transferred on to the nitrocellulose membrane employing the semi-dry trans-blot turbo apparatus (BioRad, USA). The blot was blocked with 5% non-fat dry milk followed by overnight incubation at 4^0^C of primary antibody (1:1000 dilution **#4903** Cell Signaling Technology) against NANOG under gentle agitation. Washing steps were carried out to remove excess primary antibody followed by one hour incubation at room temperature of peroxidase-labelled secondary antibody (1:2000 dilution). The signal was detected using ECL Clarity System (BioRad) using the BioRad UV gel documentation system. The ImageLab software was used to acquire and analyze the data.

### 2.7.4. Expression analysis using flow cytometer

To analyze the expression of various genes corresponding to stemness, FACS was performed. Briefly, the OCSCS and BCSCs treated with 0.6mM and 0.3mM sulfasalazine, respectively, were washed with ice-cold PBS twice and incubated with primary antibodies (OCT-4 and NANOG) for 30 mins at 4^0^C. After incubation, the cells were washed and suspended in FITC-conjugated secondary antibodies. The cells were washed with ice-cold PBS and expression was analyzed using flow cytometry.

### 2.8. Ferrozine assay

To determine the intracellular iron levels, ferrozine assay was performed. Briefly, cells were treated with 100uM ferric ammonium citrate (FAC) prior to drug targeting. After treating the cells with respective concentrations of SAS (0.6mM for OCSCs and 0.3mM BCSCs) cell lysate was isolated by adding 50mM NaOH to the treated cells after washing the cells with ice cold PBS. The solution was kept on shaker for 2 hours at room temperature and the cell lysate is collected into 1.5mL Microcentrifuge tube. The absorbance of the cell lysates were measured at 562nm and the concentration of the intracellular ferritin was derived through a standard graph.

### 2.9. Assessment of intracellular glutathione levels

Intracellular glutathione (GSH) levels were assessed using cellular GSH assay kit (CellSignaling Technologies) and the GSH levels were quantified as per the manufacturer's instructions. Briefly, cell lysate was isolated from control and drug treated CSCs were by adding digitonin lysis buffer. The solution was incubated for 5 mins at room temperature and then centrifuged for 15 mins at 14,000 rpm at 4^0^C. The supernatant was used for measuring the GSH levels by measuring the fluorescence at 380/460nm excitation/emission wavelength using a spectrofluorometer (with the help of facilities in IIT Hyderabad). Intracellular GSH levels were quantified using the standard graph.

### 2.10. Cell cycle analysis

To determine at which stage the cell has been inhibited to undergo cell division, we performed cell cycle analysis using a flow cytometer. Briefly, drug-resistant oral and breast cancer cells are treated with sulfasalazine. After 24 hours of incubation, cells were trypsinized and pelleted down. The pellet was then washed with 1X PBS twice. After completing the washing steps, the pellet is suspended in 50µL of 100µg/mL RNase and 200µL of 50µg/mL Propidium Iodide and immediately analyzed cells using a flow cytometer.

### 2.11. Cell apoptosis analysis

To analyze the percentage of cells that underwent cell death after drug treatment, cell apoptosis analysis using a flow cytometer was performed. Briefly, after incubation of the drug-resistant oral and breast cancer cells, the cells were trypsinized and pellet down to form a single-cell suspension. These cells were used for apoptosis analysis along with the Abcam Annexin-V FITC Apoptosis Staining/Detection kit. The pellet was resuspended in 500µL 1X binding buffer, 5µL Annexin V-FITC, and 1µL SYTOX green dye. The tubes were then incubated for 10 mins at room temperature in a dark atmosphere. After incubation, the cells were analyzed.

### 2.12. Cellular ROS assay

Cellular ROS plays a vital role in both cancer and CSCs to induce cell death by elevating oxidative stress. Cellular ROS assay was performed as previously-described. Briefly, drug-treated and control ALDH1A1 UCSF-OT-1109 and MCF-7 cells were washed with 1XPBS and fixed with 70% ethanol for 30 mins. After the cells were fixed, they were stained with 100µM 2′,7′-Dichlorofluorescin diacetate DCF-DA (Sigma Aldrich) for 30 mins, washed with PBS, and counter stained with 30µg/mL Hoechst 33342 for 5 mins at RT. After incubation, the cells were washed with 1XPBS to remove extra stain and background noise. The images were then captured using EVOS FL fluorescent microscope (Invitrogen).

### 2.13. Statistical analysis

All the experiments were done in duplicates and the data is presented in mean values. Student t-test is performed to compare the difference between number of spheroids formed in each generation. Pearson co-efficient and student t-test were done to measure the significance for the generated spheroids. P-value < 0.05 was considered to be statistically significant.

## 3. Results

### 3.1. UCSF-OT-1109 and MCF-7 cells express ALDH1A1 expression on their cell

**Figure 1:**
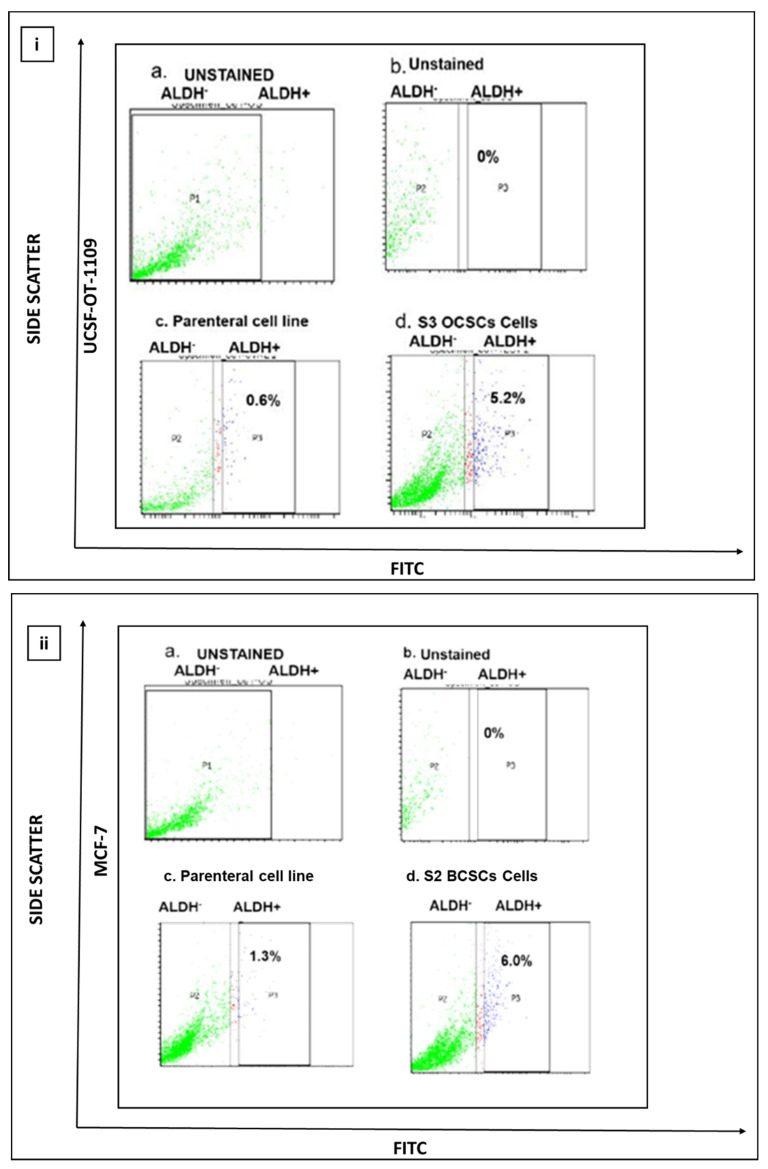
** (i) and 1 (ii). Representative pictures of CSCs present in UCSF-OT-1109 and MCF-7 cell lines** respectively**.** Elevated expression of cytoplasmic marker ALDH1A1 reflects the stemness potential of a tumor cell. Hence, to quantify the presence of ALDH1A1 marker, ALDEFLOUR assay was performed and the data was acquired using flow cytometer at 488nm excitation wavelength and 530nm emission wavelength. The sorting gates were established using the negative control. In figure 1 (i) UCSF-OT-1109 showed an increase of ALDH1A1 population from 0.6% to 5.2% with 8.6-fold change while an increase from 1.3% to 6% with 4.6-fold change in MCF-7 cells. Figures 1 (i) and (ii) (a) and 1 (i) and (ii) (b) represents unstained cells; figure 1 (i) and (ii) (c) represents ALDH1A1 parental cell lines UCSF-OT-1109 and MCF-7 respectively; Figure 1 (i) and (ii) (d) represents S3 and S2 generations of UCSF-OT-1109 and MCF-7 CSCs respectively.

Previous studies suggest that ALDH activity, represented by Aldefluor positive status, is a good indicator of tumor cell lines, particularly breast and oral, that exhibit stem cell properties, such as self-renewability, oncogenesis, and metastasis. Quantification of presence of ALDH1A1 marker suggested that both oral and breast cancer cell lines Unstained cells were considered as negative control and were used to establish the sorting gates. The cells comprising the ALDH marker are termed to be ALDH1 +ve cells (right quadrant), while the cells lacking ALDH1A1 are termed to be ALDH1 -ve cells (left quadrant). Flow cytometer analysis of ALDEFLOUR assay revealed that the UCSF-OT-1109 cell line possesses 0.6% of ALDH1A1 +ve OCSCs while the MCF-7 cell line has 1.3% ALDH1A1 +ve CSCs. **Figure 1 (a-c)** represents the levels of ALDH1A1 +ve CSCs in UCSF-OT-1109 and MCF -7 cancer cell lines, respectively.

### 3.2. Both OCSCs and BCSCs efficiently formed spheroids

**Figure 2:**
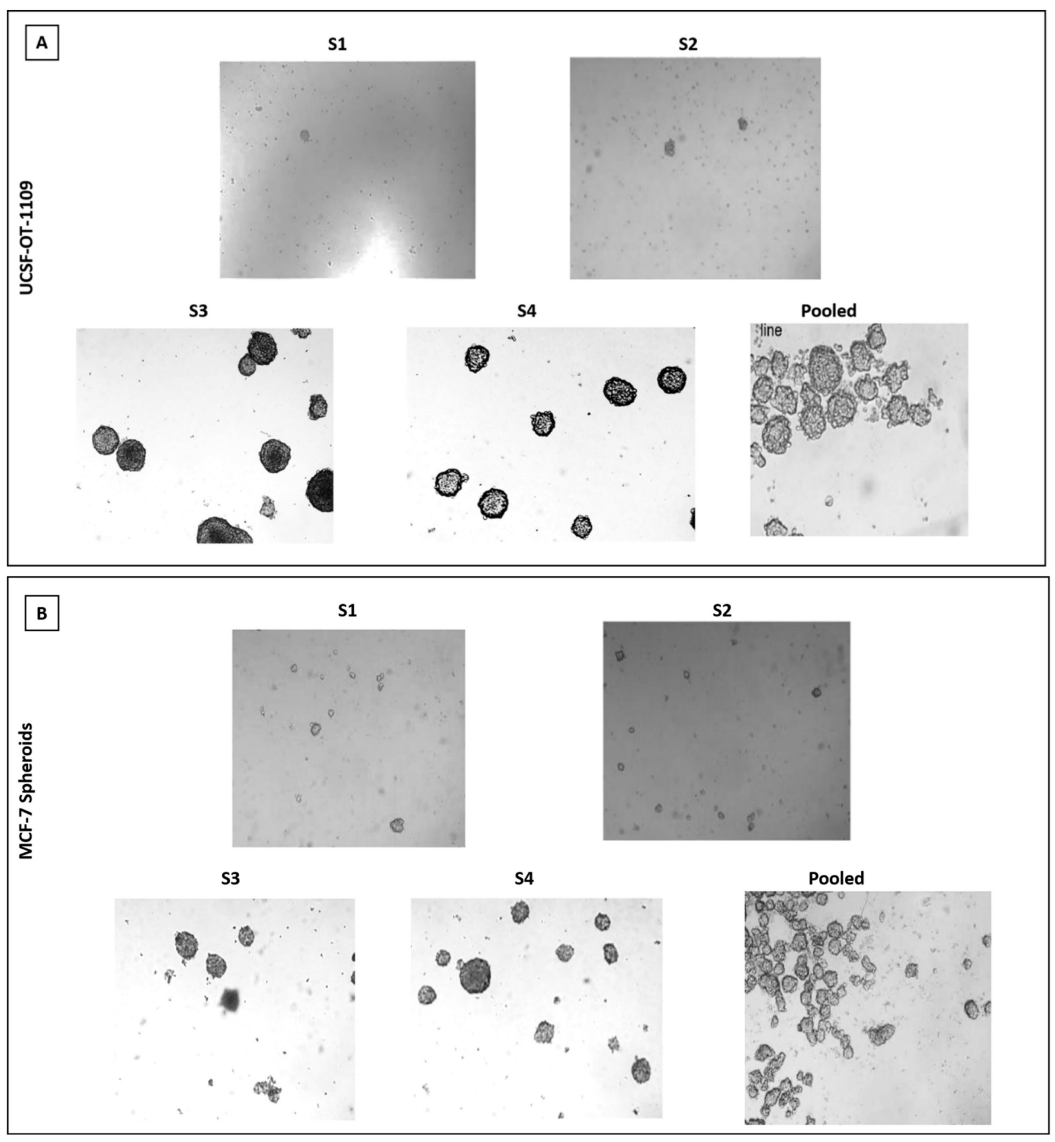
** (A) and 2 (B). Representative pictures of Spheroids formed by ALDH1A1/UCSF-OT1109 cells and ALDH1A1/MCF-7 cells respectively.** The ability to form spheroids is one of the characteristic features of cancer stem cells. Hence the ability to form spheroids was assessed by growing the primary spheroids for several generations. Briefly, the primary spheroids were enzymatically digested and plated. This was repeated for several generations until the spheroids were formed and before each enzymatic digestion the total number of spheroids was counted and tabulated. Spheroid formation assay revealed that oral CSCs potentially formed spheroids till third generation with gradual deterioration in number and size of the spheroids, while in breast CSCs only till second generation increase in size and number of spheroids has been observed. There is an increase in size of oro spheres and mammospheres with respect to the number of passages. The spheroid formation ability of ALDH1A1 OCSCs and BCSCs was observed and the pictures were captured at 10x magnification using microscope. (p-value < 0.05).

Breast epithelial, breast cancer and oral cancer spheroids were formed by enzymatic dissociation followed by plating the dissociated parental cells. The ALDH1A1 population was termed as 'S1', and the subsequent spheroids obtained by passaging the cells for a period of nine days followed by enzymatic dissociation were termed as S2, S3, and so on. The present study demonstrated that ALDH1A1-/low cells and MCF-10A cells fail to generate spheroids (supplementary figure). As demonstrated by Jayanta Debnath et, al. (2003), in our present study we observed tumor cell aggregates representing acinus-like spheroids with hollow lumen. We could observe very few cell aggregates till S2 generation after which only single cells with vacuolar formation were observed. (supplementary figure) While in ALDH1A1 cells with increasing passages, both UCSF-OT-1109 (OCSCs) and MCF-7 (BCSCs) spheroids showed a dynamic increase in the number of SFUs from ~0.6% in S1 generation to ~5.2% in S3 generation followed by a gradual reduction in S6 generation while it ranged between ~1.3% in S1 generation to ~6.0% in S2 second generation followed by a gradual reduction in S7 generation respectively **(Figure 1, d)** after which no spheroids were detected **(Figure 2).**

### 3.3. Both OCSCs and BCSCs formed spheroids with increase in passage

**Figure 3:**
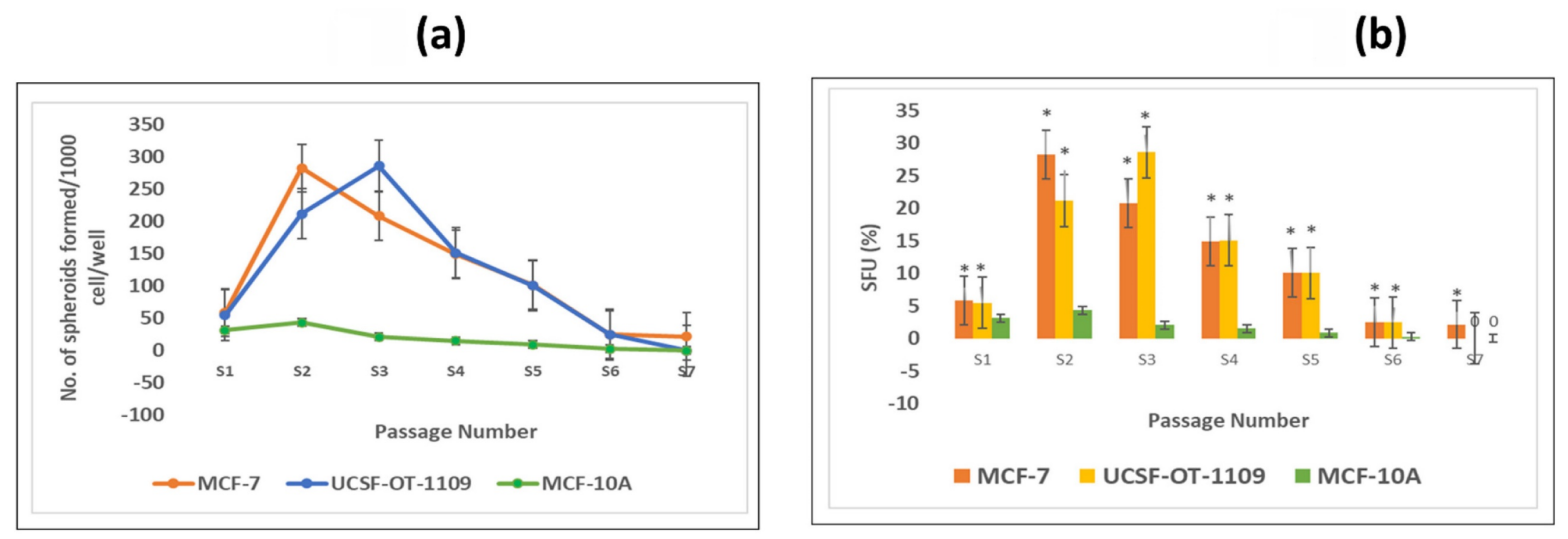
** Representative pictures of spheroids formed by ALDH1A1 CSCs isolated from MCF-7 and UCSF-OT-1109 cell lines.** To investigate the efficiency of spheroid formation by ALDH1A1 CSCs isolated from MCF-7 and UCSF-OT-1109 cells, the total number of spheroids formed during each generation was counted and the spheroid forming efficiency was calculated. Figure 3 (a) represents the total number of spheroids formed during each generation and Figure 3 (b) represents the spheroid forming efficiency of both the cell lines in each generation. Student t-test has been performed to know the statistical significance, the number of spheroids formed is statistically significant with a p-value < 0.05.

To check the self-renewability of both OCSCs and BCSCs in comparison with normal breast epithelial cells (MCF-10A), a serial sphere formation assay was performed. The obtained results suggested that the self-renewal potential of both OCSCs and BCSCs gradually increased till S3 and S2 generation respectively with logarithmic deterioration of the self-renewal ability, however, no further spheroid formation was detected in S6 and S7 generations, respectively** (Figure 3 a)**. On the other hand, due to very low ALDH1A1 cell population in MCF-10A cells, these could not form spheroids as formed by CSCs. Also, the number of tumor spheroids formed in each passage of both the CSCs was counted. As shown in **Figure 3b,** there is a gradual increase in the number of spheroids from 5.4% in S1 generation to 28.5% in S3 generation of CSCs derived from UCSFOT-1109. In the case of BCSCs derived from MCF-7 cell lines, a gradual elevation of the number of spheroids from 5.8% in S1 generation to 28.2% in S2 generation with a gradual decline in the number of spheroids has been observed. The results show that UCSF-OT-1109-derived CSCs showed an increase in SFUs till the third generation, while MCF-7-derived CSCs were able to form SFUs till the second generation, suggesting UCSF-OT-1109 exhibits more stem cell renewal potential when compared with MCF-7.

### 3.4. SAS induced cytotoxicity at different concentration in both OCSCs and BCSCs

**Figure 4:**
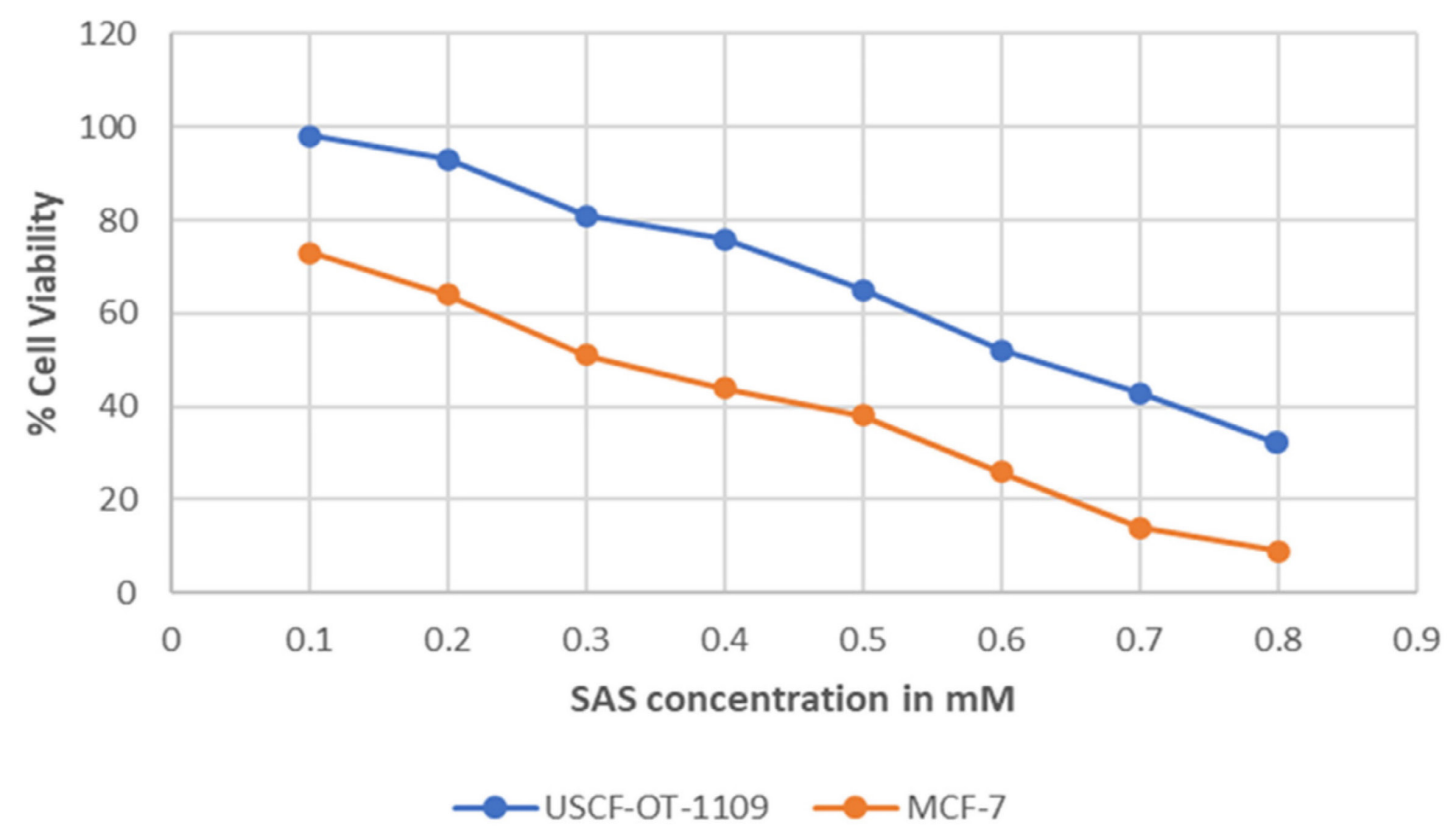
** Graphical Representation of the percent of viable cells after treating with increased concentration of SAS.** Cell viability represents the concentration of the drug at which the cell undergoes death. IC50 value represents the concentration of a certain drug that could induce cytotoxicity in half of the total population. As suggested in the figure, SAS induced a dose-dependent decrease in cell viability with an IC50 concentration of 0.6mM for UCSF-OT-1109 and 0.3mM MCF-7 respectively (p-value < 0.05).

MTT assay was performed on UCSF-OT-1109 and MCF-7 cell lines using sulfasalazine to determine the IC50 and the cytotoxic effects in the cells. Obtained results indicate a dose-dependent decrease in cell viability when the cells were treated for 24 hours. The obtained IC50 absorbance was used to calculate the concentration of the drug. We obtained IC50 values of around 0.6Mm for UCSF-OT-1109 cell lines while it is 0.3Mm for MCF-7 cells treated with sulfasalazine **(Figure 4)**. The aim of the present experiment is to initially validate whether the chosen drug is inducing cytotoxicity to the cells or not.

### 3.5. SAS treatment induced ferroptosis effectively in both OCSCs and BCSCs

**Figure 5:**
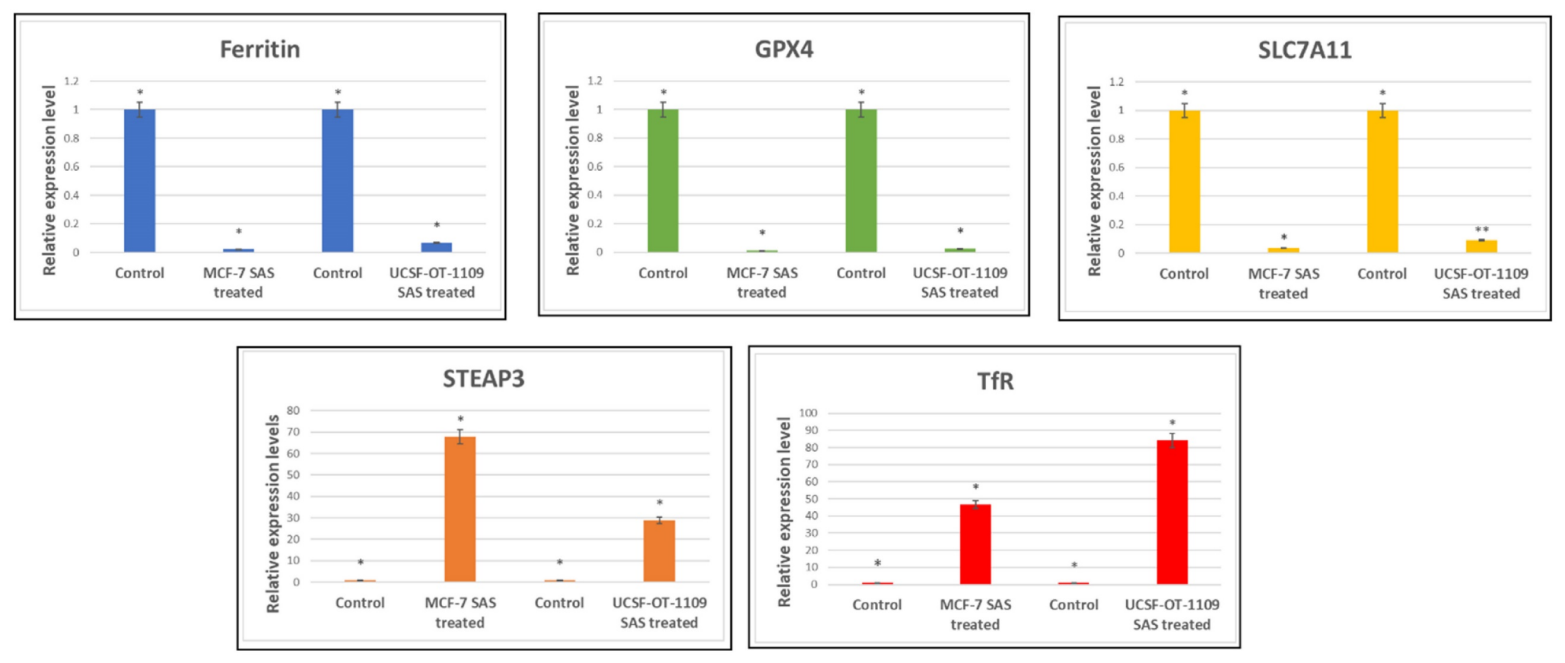
** Expression profile of genes involved in ferroptosis** Ct values derived from RT-PCR were analysed using LivakMode and the obtained data suggests that the ferroptotic markers with oncogenic activity (Ftn, SLC7A11, and GPx4) were downregulated. SAS is a known pharmacological inhibitor of SLC7A11. RT-PCR data validated that SAS inhibited SLC7A11 which is accompanied by reduced levels of GPx4 expression which is the key regulator for lipid ROS scavenging. SLC7A11 is the master regulator of glutathione synthesis which serves as co-factor for GPx4. As shown in the above figure, downregulation of SLC7A11 resulted in low glutathione levels and hence reduced expression of GPx4. On the other hand, SAS also induced ferritin degradation. While ferroptotic markers serving as tumor suppressors (TfR and STEAP3) have been significantly upregulated. P-value <0.05 is represented as * in the figure. While ** represents non-significance. (p-value < 0.05).

**Figure 6:**
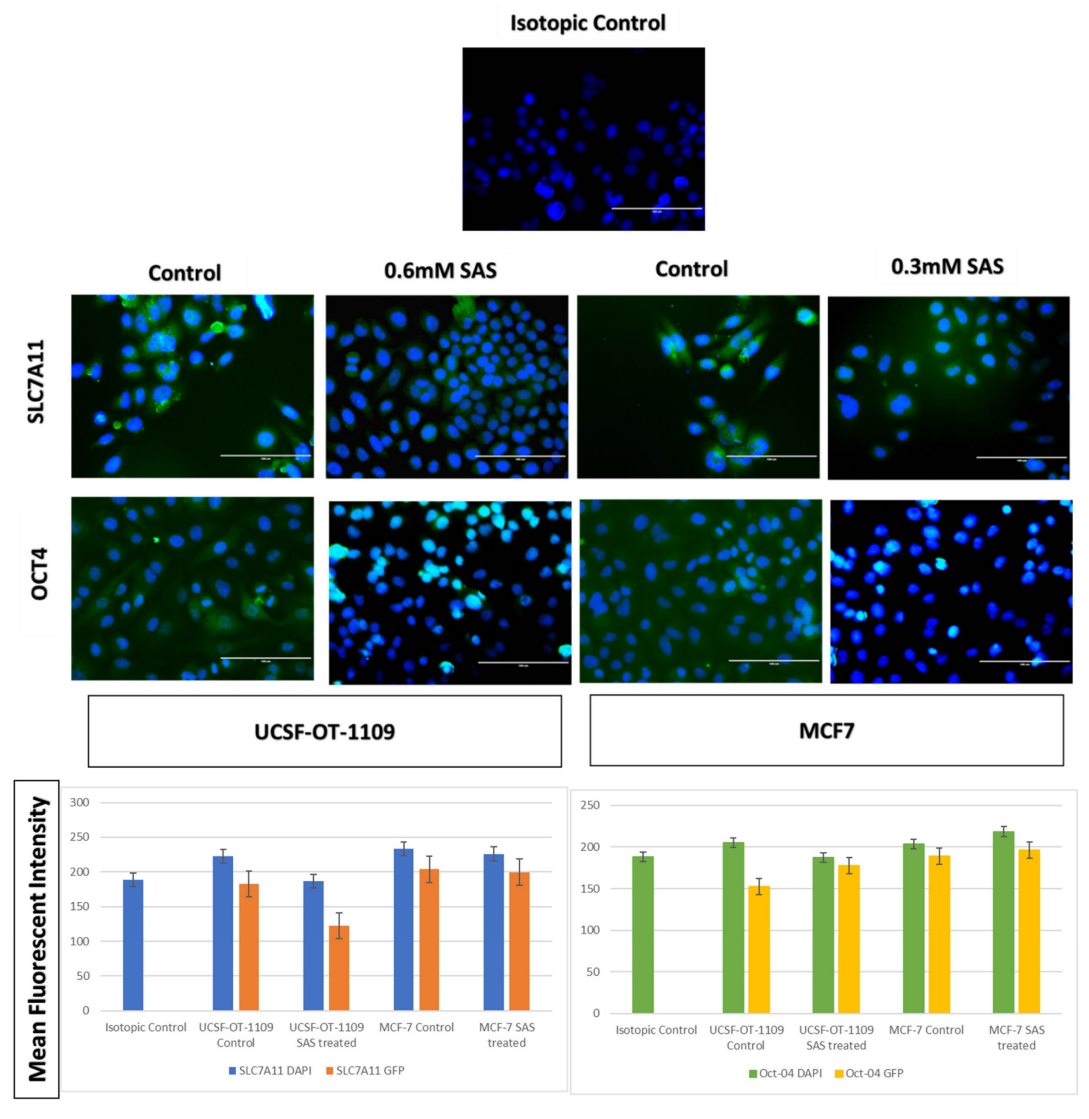
** Respective gene expression profile of SLC7A11 and OCT4 of OCSCs and BCSCs obtained by Immunofluorescence.** Cells seeded on coverslips were stained with SLC7A11 antibody, secondary antibody conjugated FITC (visualized in green color), counter stained with DAPI (visualized in blue color). As depicted in the figure, untreated cells showed bright green fluorescence on the plasma membrane, while treated cells showed light green fluorescence The mean fluorescent intensity represented in the graph suggests decrease in the expression of both SLC7A11 and OCT4 in treated samples when compared to the untreated controls. The mean fluorescent intensity represented in the graph suggests decrease in the expression of both SLC7A11 and OCT4 in treated samples when compared to the untreated controls (p-value < 0.05).

**Figure 7:**
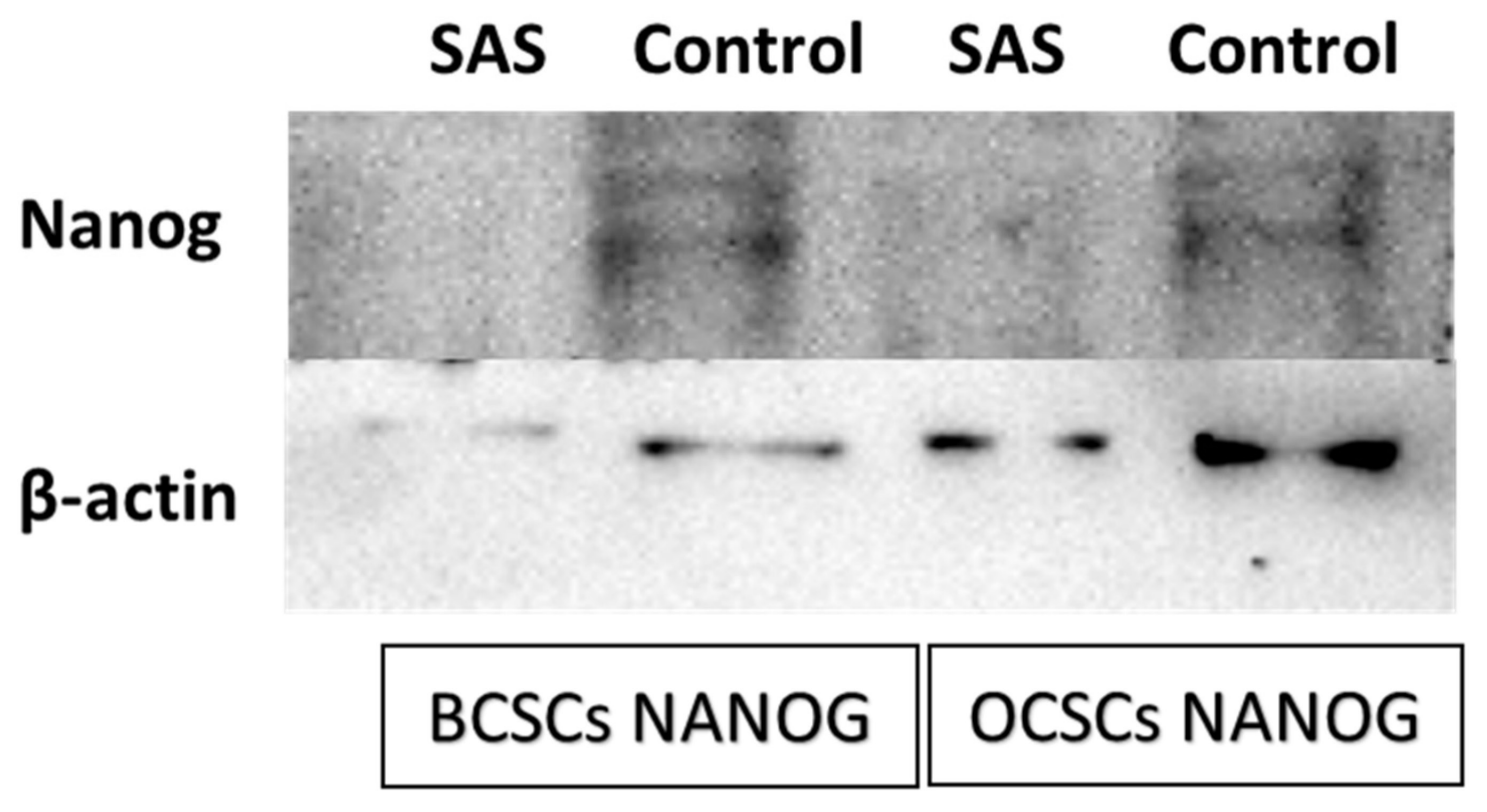
** Western Blot analysis to determine the expression of NANOG protein in both BCSCs and OCSCs.** NANOG protein expression by both BCSCs and OCSCs treated with a respective concentration of SAS was confirmed by Western blot analysis showing 40 kDa band of NANOG protein. (p-value < 0.05).

**Figure 8:**
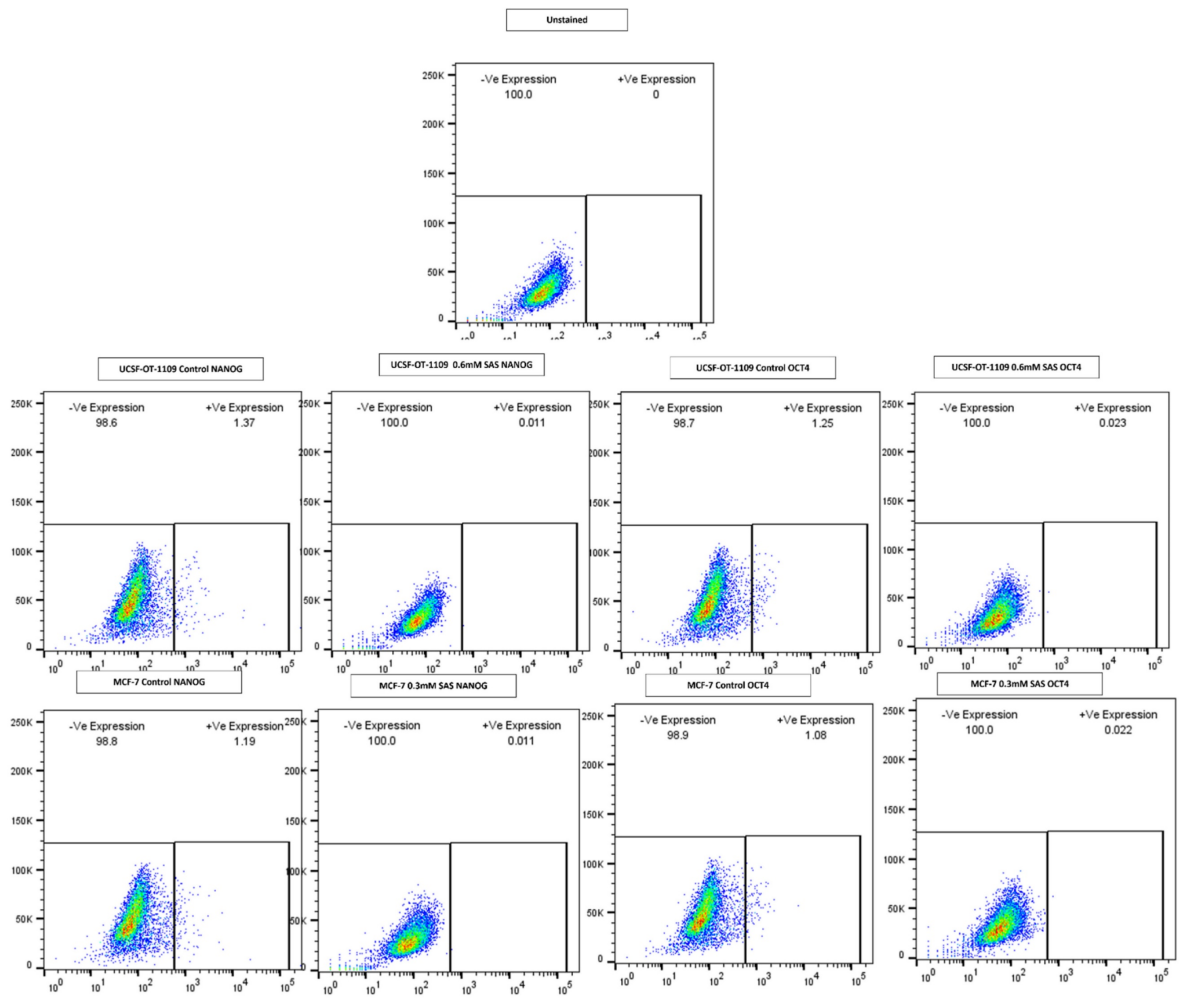
** Gene expression profile of stem cell marker genes of OCSCs and BCSCs obtained by flow cytometry analysis.** Depletion of NANOG and OCT4 a master gene that regulates stemness in CSCS ALDH1A1 expressing CSCs after SAS exposure showed a drastic decrease in the expression of NANOG from 1.37% to 0.011%; 1.19% to 0.011% in OCSCs and BCSCs. While a drastic decrease in OCT4 expression from 1.25% to 0.023%; 1.08% to 0.022%.

After exposing the ALDH1A1 CSCs with the ferroptosis-inducing drug sulfasalazine (0.6Mm for OCSCs and 0.3Mm for BCSCs), RT-PCR and IF studies were carried out to analyze the expression of various ferroptosis markers. RT-PCR analysis suggested that negative regulators of ferroptosis, such as GPx4, SLC7A11, and ferritin, were downregulated while TfR and STEAP-3, which are regarded as positive modulators of ferroptosis, have been upregulated **(Figure 5)**. To counter check RT-PCR results, IF was performed to evaluate the expression of SLC7A11 and OCT4 markers. As shown in **Figure 6**, SAS treatment reduced the expression of SLC7A11 as well as OCT4 in both OCSCs and BCSCs when compared to untreated control group. In contrast to the ferroptosis markers, flow cytometer analysis revealed a gradual decrease in the percentage of stem cell markers such as NANOG, and OCT-4, when compared with the control cell lines (untreated cells). Western blot analysis suggested a very low concentration of NANOG in control cells, the expression of the same was completely inhibited when both BCSCs and OCSCs were treated with their respective IC50 concentrations suggesting that SAS effectively suppressed the expression of NANOG **(Figure 7)**. As shown in **Figure 8,** the percentage of NANOG has decreased from 1.37% to 0.011% and 1.19% to 0.011% in OCSCs and BCSCs respectively. Similar results have been observed for OCT4 expression with a gradual decrease from 1.25% to 0.023% in OCSCs and 1.08% to 0.022% in BCSCs suggesting that SAS affected the self-renewal and pluripotent ability of both OCSCs and BCSCs and made these cells sensitive to ferroptosis. All the results obtained from RT PCR, Immunofluorescence, Western blot, and Flow cytometry suggest that SAS treatment effectively induced ferroptosis coupled with reduction in stemness.

### 3.6. SAS treatment resulted in drastic decrease of intracellular ferritin levels in both OCSCs and BCSCs

**Figure 9:**
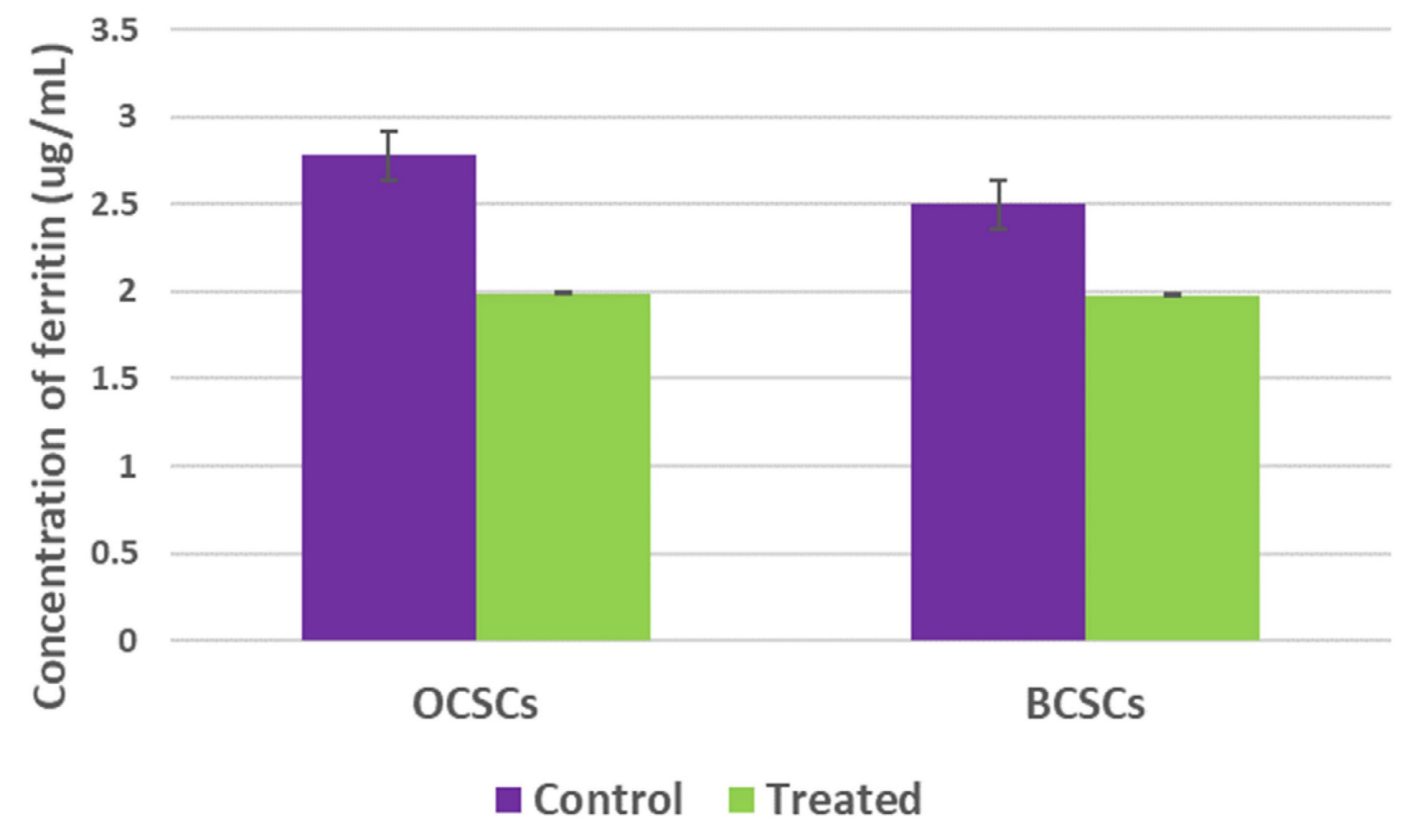
** Graphical representation of intracellular ferritin levels.** Ferritin is one of the important protein that plays a double-edge sword role in regulating tumor cell proliferation, stemness, and death. In order to assess the alteration of intracellular ferritin levels upon SA treatment both the cell lines with their respective controls were used to perform ferrozine assay. Obtained results demonstrated that SAS induced ferroptosis resulted in reduction of intracellular ferritin levels. (p-value < 0.05).

Ferritin is the most vital protein that stores bulk iron and mediates tumor cell proliferation. Numerous studies have demonstrated that high ferritin levels represent worst cancer prognosis. Apart from this, ferritin degradation is one of the hallmarks of ferroptosis induction. RT-PCR analysis suggested downregulation of ferritin gene expression. However, to counter-check the obtained results, ferritin protein quantification was performed using a ferrozine assay. As shown in **Figure 9**, intracellular ferritin levels were drastically reduced from 2.78ug/mL in untreated OCSCs to 1.99ug/mL in OCSCs treated with SAS. Similarly, BCSCs also showed reduced intracellular ferritin levels from 2.64ug/mL in control to 1.98ug/mL SAS treated BCSCs suggesting that SAS mediated ferritin degradation thereby inducing ferroptosis.

### 3.7. SAS treatment resulted in drastic decrease of intracellular reduced GSH levels in both OCSCs and BCSCs

**Figure 10:**
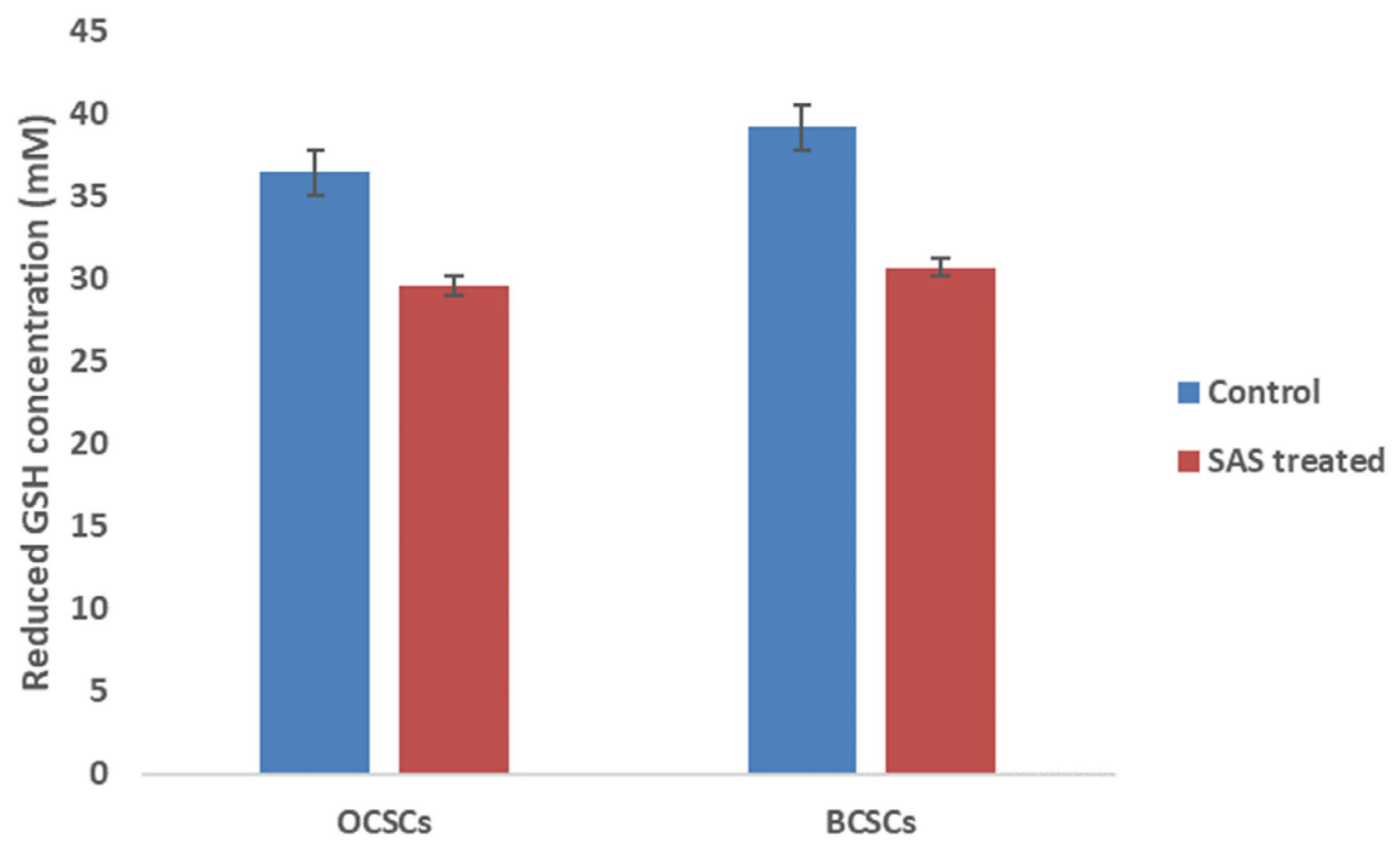
** Graphical representation of intracellular reduced GSH levels.** Reduced GSH inside the cells is the critical regulator that protects both cancer and CSCs from lipid ROS by converting the generated lipid ROS to lipid hydroxides. Hence, reduced intracellular GSH was quantified in both CSCs before and after treatment with SAS. The results indicated that SAS-induced ferroptosis is mediated through depletion of reduced intracellular GSH which resulted in the accumulation of lipid ROS. (p-value < 0.05).

Reduced GSH levels play a critical role in protecting both cancer and CSCs from ferroptosis by serving as lipid ROS scavenger molecule. GSH serve as co-factor for glutathione peroxidase-4 (GPx4) enzyme which catalyzes the conversion of lipid peroxides to lipid hydroxides thereby preventing iron-mediated cell death. Although gene expression profile suggested reduced expression of GPx4, to counter check the obtained result we quantified the protein level of GSH which is the regulator of GPx4 enzyme activity. Interestingly, we observed low levels of reduced GSH levels in both the cell lines when compared to control. As shown in **Figure 10**, GSH levels were drastically reduced from 36.4Mm to 29.4Mm in OCSCs treated with SAS; while in BCSCs a reduction from 39.1Mm in control to 30.6Mm in SAS treated cells was observed suggesting that SAS a SLC7A11 inhibitor induced ferroptosis by lowering reduced GSH levels in both CSCs.

### 3.8. SAS treatment effectively halted cell proliferation in both OCSCs and BCSCs

**Figure 11:**
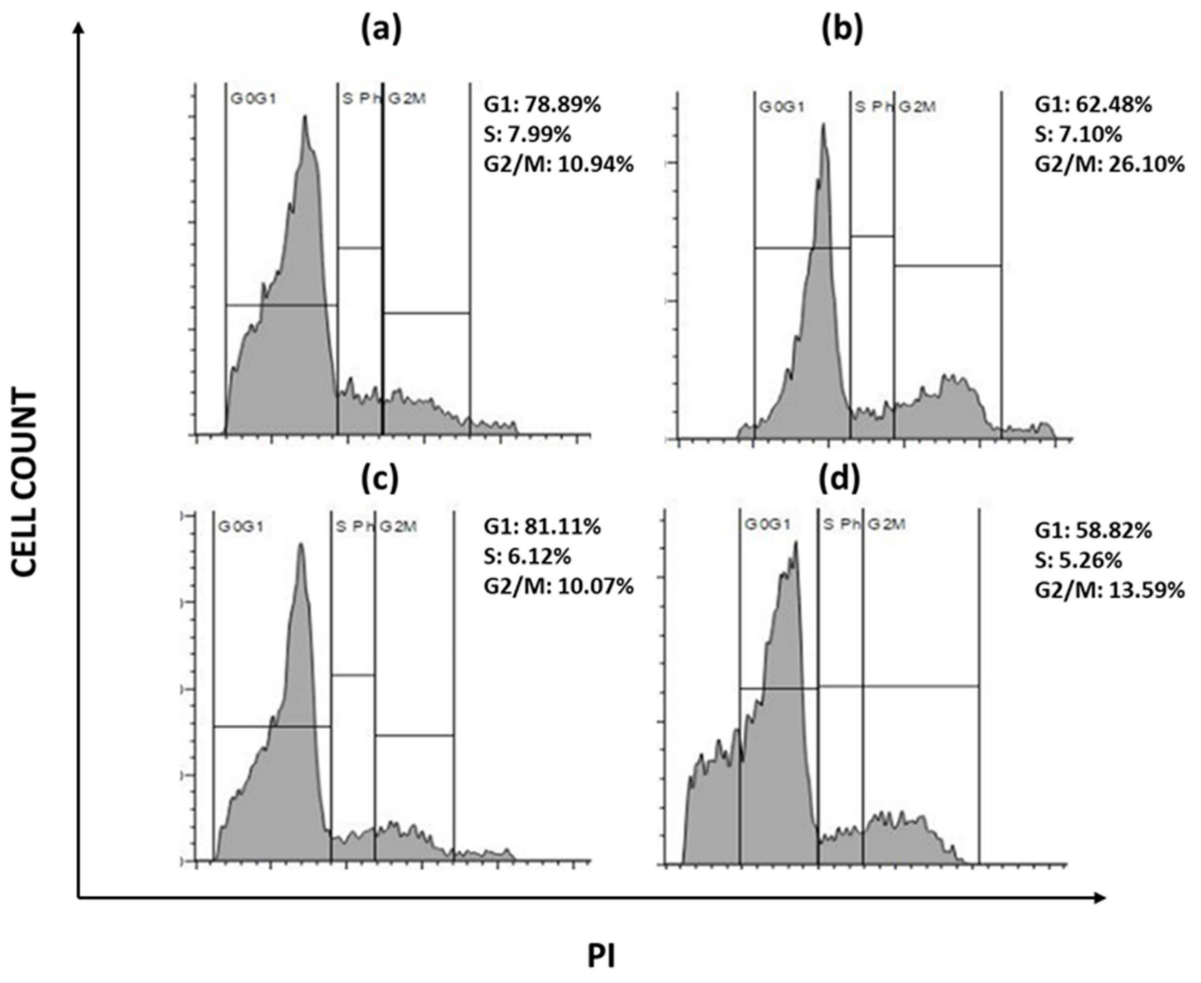
** Flow cytometry analysis representing the cell cycle arrest.** s(a) represents USCF-OT-1109 CSCs control; (b) represents USCF-OT-1109 CSCs treated with 0.6mM SAS; (c) represents MCF-7 CSCs control; (d) represents MCF-7 CSCs treated with 0.3mM SAS. As shown in Figure 6, there is a rise in percentage of cells from 10.94 to 26.10 and 10.07 to 13.59 in the G2/M phase of OCSCs and BCSCs respectively, suggesting that the cells have accumulated in G2/M phase indicating SAS induced cell cycle arrest during the G2/M progression. (p-value < 0.05).

Due to the presence of various growth factors that are constantly expressed, tumor cells have irregular cell proliferation. The ability of a compound to halt cell cycle progression at specific checkpoints leading to sensitization of tumor cells to cell death is regarded as hallmark of novel antineoplastic drugs. To further investigate the effects of sulfasalazine in inducing ferroptosis, their effect on the cell progression has been checked. Briefly, cellular DNA of ALDH1A1 CSCs were treated with PI and the progression of cells into various phases of cell cycle has been monitored. The obtained data revealed that the cells have been accumulated in the G2/M phase of the cell cycle in both the groups with an increase from 10.9% to 26.1% in the G2/M phase in OCSCs, while an increase from 10.7% to 13.59% is observed in BCSCs treated with their respective IC50 concentration for 24 hours **(Figure 11)**. Since CSCs are highly metastatic and proliferative, it is important to know whether the chosen drug could arrest the cells during their proliferation. Hence it is important to check the cell cycle arrest. The obtained data suggests that SAS induced G2/M phase arrest in both OCSCs and BCSCs.

### 3.9. SAS treatment effectively induced apoptosis in both OCSCs and BCSCs

**Figure 12:**
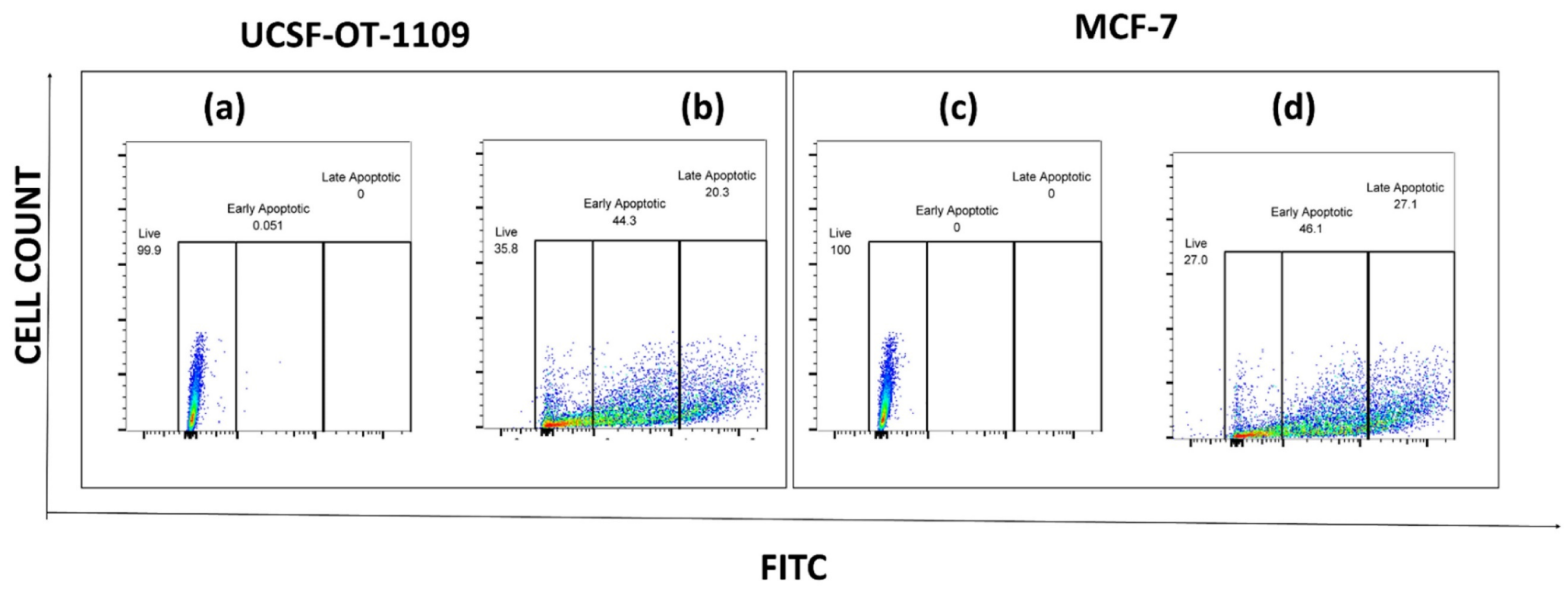
** Flow cytometry analysis representing the total percentage of cells that have undergone apoptosis.** Flow cytometry analysis of the percent of apoptotic cells in ALDH1A1 CSCs before and after AS treatment. ALDH1A1 CSCs were exposed to the IC50 concentration of SAS and the apoptotic cells were quantified by staining the cells with Annexin-FITC and STOX green. The stained cells were analyzed for apoptosis using a flow cytometer. (a) represents USCF-OT-1109 CSCs control; (b) represents USCF-OT-1109 CSCs treated with 0.6mM SAS; (c) represents MCF-7 CSCs control; (d) represents MCF-7 CSCs treated with 0.3mM SAS. As shown in Figure 10, there is a decrease in the percentage of live cells from 99.9 to 35.8 and 100 to 27 accompanied by an increase in the percentage of early apoptotic cells from 0.051 to 44.3 and 0 to 46.1 in OCSCs and BCSCs respectively, suggesting that SAS treatment induced early apoptosis. (p-value < 0.05).

To further analyze the apoptotic cells generated as a result of sulfasalazine treatment, an apoptosis analysis was performed. After exposure of cells to sulfasalazine, they were treated as per the manufacturer's protocol. Obtained data suggests that both OCSCs and BCSCs treated with the respective concentrations of sulfasalazine (0.6Mm for OCSCs and 0.3Mm for BCSCs) showed a significant increase in apoptotic cell populations. The viable OCSC populations have been decreased from 99.9% to 35.8% in cells treated with 0.6Mm sulfasalazine. However, a pronounced elevation from 0% to 44.3% in the annexin-V+/SYTOX- quadrant, representing early apoptosis, has been detected in OCSCs treated with 0.6Mm sulfasalazine. Similar results were obtained in BCSCs treated with 0.3Mm sulfasalazine, where the viable cell population was decreased from 100% to 27%, with an increase in cell population from 0% to 46.1% in the annexin-V+/SYTOX- quadrant, which indicates early apoptosis **(Figure 12)**.

### 3.10.SAS treatment resulted in elevation of intracellular ROS levels in both OCSCs and BCSCs

**Figure 13:**
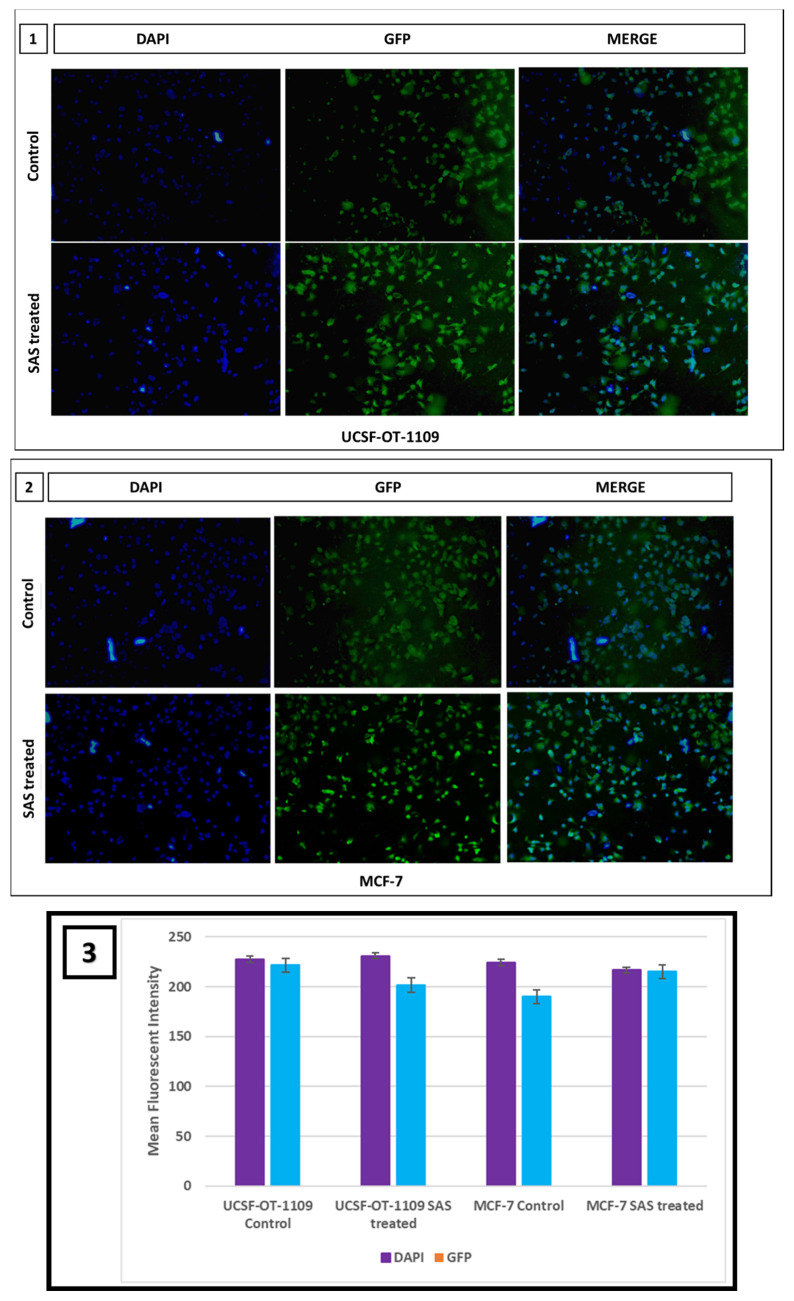
** Fluorescent microscopic images representing cellular ROS generated in UCSF-OT-1109 and MCF-7 cells expressing ALDH1A1 marker.** CSCs produce very little ROS to sustain the tumor microenvironment. Fluorescent images of control and SAS-treated cells stained with DCF-DA and Hoechst suggest that SAS elevated ROS which is indicated by the presence of bright green fluorescence with piknotic nuclei (visualized in blue color). While control cells show large nuclei with little green fluorescence. Figure 13 (1) indicates representative pictures of ROS generated in OCSCs; Figure 13 (2) indicates representative pictures of ROS generated in BCSCs. Figure 13 (3) represents the mean fluorescent intensity suggests decrease in the expression of both SLC7A11 and OCT4 in treated samples when compared to the untreated controls. (p-value < 0.05).

Intracellular ROS levels were measured using 2′, 7′-dichlorofluorescein diacetate (DCF-DA) fluorescence and were counterstained with Hoechst nuclear stain to assess the effect of SAS on generating cellular lipid ROS. To sustain the inevitable tumor microenvironment, CSCs produce little ROS that enables these cells to maintain their proliferation. Inverted fluorescence microscopy revealed, little green fluorescence accompanied with bright blue fluorescence in control group while significant DCF fluorescence accompanied with pyknotic nuclei was observed in SAS treated group, indicating the generation of intracellular ROS in both oral and breast CSCs treated with 0.6Mm and 0.3Mm sulfasalazine, respectively in a dose dependent manner **(Figure 13)**.

## 4. Discussion

Cancer is a considered a metabolic disorder that is characterized by the dysregulation of various metabolic pathways. For instance, dysregulated iron metabolism is one of the hallmarks of cancer leading to the worst survival outcome 18. Rooting evidence suggests that both cancer and CSCs accumulate iron in large quantities and store it in the form of ferritin. Ferritin reserves in CSCs confer resistance to therapy, making them the root cause for cancer recurrence and relapse 19, 20. CSCs accompany a small population of cells within the tumor. They are also regarded as 'tumor-initiating cells' or 'tumorigenic cells.' Cancer stem cells (CSCs) are similar to that normal stem cells (NSCs) in the ability to self-renew and differentiate 21, 22. The difference between NSCs and CSCs is that the differentiation and proliferation of NSCs is highly regulated, while CSCs lose their regulation in controlling the cell cycle 23. The cancer stem cell model proposed that CSCs that form a subset of tumor cells, are ultimately responsible for tumor initiation, progression, and recurrence 24. Tumor metastases and resistance to therapies are directly associated with CSCs. The first compelling evidence for the presence of CSCs came into light around 1997 by the discovery of Bonnet and Dick. There are many markers that could differentiate CSCs from NSCs such as CD44, CD133, CD24, EpCam, ALDH1A1, etc.; these markers are highly expressed in wide types of cancer 25-27.

Although various treatment strategies are being clinically practiced, none of them have shown to be effective in targeting CSCs. Taking a clue that CSCs are rich in ferritin, targeting ferritin degradation might induce a novel cell death pathway that elevates lipid ROS production through Fenton reactions in a process called ferroptosis 28-30. Ferritin is an iron-storage protein that reserves iron. Cancer and CSCs rely on iron to sustain the harsh tumor-microenvironment. For instance, ribonucleotide reductase is an iron containing enzyme that catalyzes the conversion of ribo nucleic acids to deoxy ribo nucleic acids, thereby permitting continuous DNA synthesis 31. On the other hand, the hypoxic environment, which is regarded as the hallmark of both cancer and CSCs, is regulated by iron bioavailability. Prolyl hydroxylases are the enzymes that degrade HIF-1α, which leads to normoxia. Interestingly, this enzyme requires iron as a co-factor for its activation. But CSCs suppress the activation of prolyl hydroxylases, thereby maintaining the hypoxic environment. Rooting evidence suggest that ferritin, when degraded, induces a novel pathway which is caspase-independent.

Ferroptosis is an iron-dependent pathway that induces tumor cell death by elevating lipid ROS catalyzed by the activation of lipoxygenase 32-34. Although CSCs are more sensitive to ferroptosis, they abscond the cell death mechanism by elevating intracellular glutathione levels (GSH) which serve as lipid ROS scavenging molecules 20, 35. SLC7A11, a cysteine glutamate antiporter formerly termed as xCT, is the major regulatory gene for elevated GSH levels. xCT imports one cysteine molecule with concomitant export of one glutamate molecule. The imported cysteine molecule is further metabolized into cystine and would be coupled with intracellular glutamate and glycine to produce GSH. The synthesized GSH serves as a co-factor to active Glutathione peroxidase 4 (GPx4) which catalyzes the conversion of lipid ROS to lipid hydroxides, thereby protecting the cells from ferroptosis 36-38. Ferritin, which usually stores iron in ferric state, would be converted into ferrous, which in turn activates various enzymes that trigger tumor cell death through induction of ferroptosis.

In the present study, the effect of SAS on inducing ferroptosis was investigated in OCSCs and BCSCs. SAS is an xCT inhibitor that inactivates SLC7A11 genes. We hypothesized that inhibition of SLC7A11 results in low GSH levels, which eventually leads to oxidative stress-induced cell death. Interestingly, results indicate that SAS treatment increased transferrin and STEAP-3 expression, while decreased ferritin, SLC7A11, and GPx-4 expression, which are the major drivers that protect the tumor cells from ferroptosis. Usually, high ferritin and GPx-4 expression serve as a shield for the CSCs to abscond ferroptosis 39-41. However, sulfasalazine, which is a potent inhibitor of SLC7A11, could degrade ferritin and elevated the transferrin receptor, thereby promoting ferroptosis. To confirm that sulfasalazine has induced ferroptosis, we also evaluated lipid ROS content, which is an indicator for oxidative stress. Analysis of lipid ROS suggested that ALDH +ve-expressing OCSCs and BCSCs have high lipid ROS when compared with control cells, which confirmed that ferroptosis has been induced in both OCSCs and BCSCs expressing ALDH1A1.

We observed that breast cancer cell lines contain distinct populations that display ALDH-/low and ALDH1A1/bright phenotypes. On the other hand, breast cancer cell lines showed an increase in the number of differentiated cells, which resulted in the generation of unfavorable niches, thereby altering their self-renewal and differentiation potential over multiple *in vitro* passages. The stem cell niche is very important for the cancer cell to regulate stemness, acquire resistance against anti-cancer drugs or radiation, and to protect the cells from undergoing cell death (22). However, when the S2 generation spheroids were treated with sulfasalazine, we observed a decreased expression of OCT-4 and NANOG, which are the major drivers of self-renewal ability in cancer stem cells, suggesting that the ferroptosis inducer, aside from inducing ferroptosis, is also capable of reducing the self-renewable potentiality of OCSCs and BCSCs.

## 5. Conclusion

After the leads that we obtained from the present study, we suggest that ferroptosis, a novel cell death pathway, could reduce the self-renewal ability, thereby reducing drug resistance in both OCSCs and BCSCs. Maintaining redox balance is vital for progression through the reprogramming process, and that xCT-mediated cystine uptake is essential for chief cell plasticity and ROS detoxification. Our data showed that SLC7A11 inhibition reduced stemness and induced cell death through inhibition of glutamate uptake. xCT gene is essential for glutamate uptake as it serves as an antiporter that influx glutamate through cysteine efflux. Since treatment with SAS inhibited the expression of xCT, glutamate uptake has been halted. Also, spectrofluorometer studies showed decrease in glutathione levels which is due to the depletion of glutamate. All these results suggest that SAS treatment led to a decrease in glutamate stores, which eventually led to the induction of oxidative stress. Although the present study demonstrated the role of SAS in inducing ferroptosis in CSCs through inhibiting xCT, our results showed a gradual decrease in ferritin gene expression, suggesting that SAS potentially degraded the store ferritin. However, the exact mechanism through which ferritin degradation has been induced was not investigated in the present study. Further research on the mechanism of ferritin degradation through SAS treatment should be investigated.

## Supplementary Material

Supplementary figures.

## Figures and Tables

**Table 1:** Tabulation of various primers used in the present study.

Gene	Forward Primer	Reverse Primer
Ferritin RNA	GCTCTACGCCTCCTACGTTT	GTGGCCAGTTTGTGCAGTTC
STEAP-3 mRNA	GCGGACCTTCAGCTGCC	GGCTACTATCGCTGTCCACC
SLC7A11 mRNA	CTGCTTTGGCTCCATGAACG	GGCAGATTGCCAAGATCTCAAG
GPX-4 mRNA	ATTGGTCGGCTGGACGAG	CCGAACTGGTTACACGGGAA
TfR mRNA	GAACTACACCGACCCTCGTG	GTGCTGTCCAGTTTCTCCGA
GAPDH	ACAGTCAGCCGCATCTTCTT	GGCAACAATATCCACTTTACC

## References

[1] Malla RR, Pandrangi S, Kumari S, Gavara MM, Badana AK (2018). Exosomal tetraspanins as regulators of cancer progression and metastasis and novel diagnostic markers. Asia Pac J Clin Oncol.

[2] Pandrangi SL, Parimella S (2021). Emerging Therapeutic Efficacy of Alkaloids as Anticancer Agents. Annals of the Romanian Society for Cell Biology.

[3] Barnum KJ, O'Connell MJ (2014). Cell cycle regulation by checkpoints. Methods Mol Biol.

[4] Chittineedi P, Pandrangi SL, Neira Mosquera JA, Sanchez Llaguno SN, Mohiddin GJ (2023). Aqueous Nyctanthes arbortristis and doxorubicin conjugated gold nanoparticles synergistically induced mTOR-dependent autophagy-mediated ferritinophagy in paclitaxel-resistant breast cancer stem cells. Front Pharmacol.

[5] Bourseau-Guilmain E, Griveau A, Benoit JP, Garcion E (2011). The importance of the stem cell marker prominin-1/CD133 in the uptake of transferrin and in iron metabolism in human colon cancer Caco-2 cells. PLoS One.

[6] Schatton T, Frank NY, Frank MH (2009). Identification and targeting of cancer stem cells. Bioessays.

[7] Borse V, Konwar AN, Buragohain P (2020). Oral cancer diagnosis and perspectives in India. Sens Int.

[8] Mehrotra R, Yadav K (2022). Breast cancer in India: Present scenario and the challenges ahead. World Journal of Clinical Oncology.

[9] Januchowski R, Wojtowicz K, Sterzyſska K, Sosiſska P, Andrzejewska M, Zawierucha P (2016). Inhibition of ALDH1A1 activity decreases expression of drug transporters and reduces chemotherapy resistance in ovarian cancer cell lines. Int J Biochem Cell Biol.

[10] Pandrangi SL (2014). Establishment and characterization of two primary breast cancer cell lines from young Indian breast cancer patients: mutation analysis. Cancer Cell International.

[11] Meng E, Mitra A, Tripathi K, Finan MA, Scalici J, McClellan S (2014). ALDH1A1 maintains ovarian cancer stem cell-like properties by altered regulation of cell cycle checkpoint and DNA repair network signaling. PLoS One.

[12] Wu W, Schecker J, Wurstle S, Schneider F, Schonfelder M, Schlegel J (2018). Aldehyde dehydrogenase 1A3 (ALDH1A3) is regulated by autophagy in human glioblastoma cells. Cancer Lett.

[13] Pandrangi SL, Chikati R, Chauhan PS, Kumar CS, Banarji A, Saxena S (2014). Effects of ellipticine on ALDH1A1-expressing breast cancer stem cells-an in vitro and in silico study. Tumour Biol.

[14] Pandrangi SL (2022). Role of dietary iron revisited: in metabolism, ferroptosis and pathophysiology of cancer. American Journal of Cancer Research.

[15] Recalcati S, Gammella E, Cairo G (2019). Dysregulation of iron metabolism in cancer stem cells. Free Radic Biol Med.

[16] Pandrangi SL, Chittineedi P, Chalumuri SS, Meena AS, Neira Mosquera JA, Sanchez Llaguno SN (2022). Role of Intracellular Iron in Switching Apoptosis to Ferroptosis to Target Therapy-Resistant Cancer Stem Cells. Molecules.

[17] Brown RAM, Richardson KL, Kabir TD, Trinder D, Ganss R, Leedman PJ (2020). Altered Iron Metabolism and Impact in Cancer Biology, Metastasis, and Immunology. Front Oncol.

[18] Mele L, Del Vecchio V, Liccardo D, Prisco C, Schwerdtfeger M, Robinson N (2020). The role of autophagy in resistance to targeted therapies. Cancer Treat Rev.

[19] Chittineedi P (2022). Analyzing the drivers of cancer relapse: hypocalcemia and iron absorption in hormone-dependent female cancers. Am J Transl Res.

[20] Chittineedi P Llaguno Concomitant Therapy of Aq. Theobroma Extract and Doxorubicin Reduces Stemness and Induces Ferroptosis in Therapeutic Resistant Cervical Cancer Cells. Journal of Carcinogenesis and Mutatgenesis.

[21] Fulawka L (2014). Cancer stem cells - the current status of an old concept: literature review and clinical approaches. Biological Research.

[22] Li Y, Wang Z, Ajani JA, Song S (2021). Drug resistance and Cancer stem cells. Cell Commun Signal.

[23] Batlle E, Clevers H (2017). Cancer stem cells revisited. Nat Med.

[24] Dean M, Fojo T, Bates S (2005). Tumour stem cells and drug resistance. Nat Rev Cancer.

[25] Xu X, Zhang X, Wei C, Zheng D, Lu X, Yang Y (2020). Targeting SLC7A11 specifically suppresses the progression of colorectal cancer stem cells via inducing ferroptosis. Eur J Pharm Sci.

[26] Horibe S, Kawauchi S, Tanahashi T, Sasaki N, Mizuno S, Rikitake Y (2018). CD44v-dependent upregulation of xCT is involved in the acquisition of cisplatin-resistance in human lung cancer A549 cells. Biochem Biophys Res Commun.

[27] Lakhanpal M, Singh LC, Rahman T, Sharma J, Singh MM, Kataki AC (2016). Study of single nucleotide polymorphisms of tumour necrosis factors and HSP genes in nasopharyngeal carcinoma in North East India. Tumour Biol.

[28] Tomita H (2016). Aldehyde dehydrogenase 1A1 in stem cells and cancer. Oncotarget.

[29] Kumar GR, Chikati R, Pandrangi SL, Kandapal M, Sonkar K, Gupta N (2013). Molecular docking and dynamics simulations of A.niger RNase from Aspergillus niger ATCC26550: for potential prevention of human cancer. J Mol Model.

[30] Mishra MN, Chandavarkar V, Sharma R, Bhargava D (2019). Structure, function and role of CD44 in neoplasia. J Oral Maxillofac Pathol.

[31] Chittineedi P, Mohammed A, Abdul Razab MKA, Mat Nawi N, Pandrangi SL (2023). Polyherbal formulation conjugated to gold nanoparticles induced ferroptosis in drug-resistant breast cancer stem cells through ferritin degradation. Front Pharmacol.

[32] Ryu MS, Duck KA, Philpott CC (2018). Ferritin iron regulators, PCBP1 and NCOA4, respond to cellular iron status in developing red cells. Blood Cells Mol Dis.

[33] Richa Gulati MNR, Venkata Satya Mahesh Kumar Metta, Santhi Latha Pandrangi (2021). Exploring the CRISPR/Cas9 System in Targeting Drug Resistant Cancer Stem Cells. Annals of the Romanian Society for Cell Biology.

[34] Proneth B, Conrad M (2019). Ferroptosis and necroinflammation, a yet poorly explored link. Cell Death Differ.

[35] Yang WS, Kim KJ, Gaschler MM, Patel M, Shchepinov MS, Stockwell BR (2016). Peroxidation of polyunsaturated fatty acids by lipoxygenases drives ferroptosis. Proc Natl Acad Sci U S A.

[36] Dixon SJ, Stockwell BR (2019). The Hallmarks of Ferroptosis. Annual Review of Cancer Biology.

[37] Doll S, Proneth B, Tyurina YY, Panzilius E, Kobayashi S, Ingold I (2017). ACSL4 dictates ferroptosis sensitivity by shaping cellular lipid composition. Nat Chem Biol.

[38] Rambatla P (2021). A Study on the Expression of CCL5, CXCR4 and Angiogenic Factors by Prostate Cancer Stem Cells. Annals of the Romanian Society for Cell Biology.

[39] Peng G, Tang Z, Xiang Y, Chen W (2019). Glutathione peroxidase 4 maintains a stemness phenotype, oxidative homeostasis and regulates biological processes in Panc-1 cancer stem-like cells. Oncol Rep.

[40] Pandrangi SL, Chittineedi P, Mohiddin GJ, Mosquera JAN, Llaguno SNS (2023). Cell-cell communications: new insights into targeting efficacy of phytochemical adjuvants on tight junctions and pathophysiology of various malignancies. J Cell Commun Signal.

[41] Bekeschus S, Eisenmann S, Sagwal SK, Bodnar Y, Moritz J, Poschkamp B (2020). xCT (SLC7A11) expression confers intrinsic resistance to physical plasma treatment in tumor cells. Redox Biol.

