# Supplementary material for: Down-Regulation of Cysteine-Glutamate Antiporter in ALDH1A1 Expressing Oral and Breast Cancer Stem Cells Induced Oxidative Stress-Triggered Ferroptosis
Source: J Cancer. 2024 Oct 7;15(19):6160–76. doi: 10.7150/jca.89429 (PMC11540493; doi:10.7150/jca.89429)
Supplement: Supplementary file 1 — Supplementary figures. [file jcav15p6160s1.pdf]

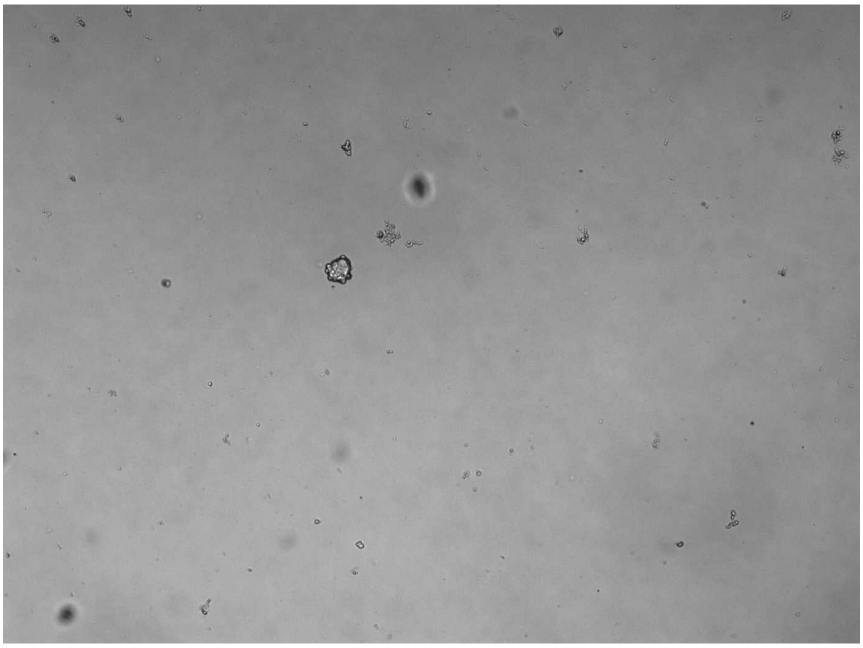

Supplementary figure 1. Representative picture of spheroid forming assay of breast normal cell line MCF-10A. As shown in the figure, MCF-10A cells formed aggregates that resemble acinus-like structures (Jayanta Debnath et, al. (2003)) suggesting that MCF-10A could not form spheroids similar to cancer cell lines.

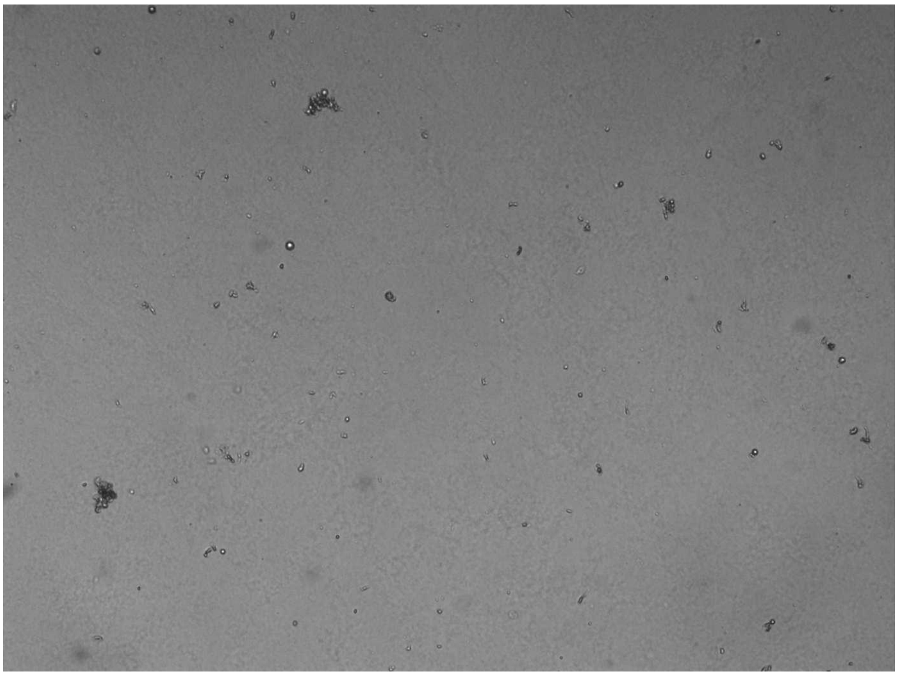

Supplementary figure 2. Representative picture of spheroid forming assay of ALDH1A1 negative cells.
